# Supplementary material for: LFA-1 nanoclusters integrate TCR stimulation strength to tune T-cell cytotoxic activity
Source: Nat Commun. 2024 Jan 9;15:407. doi: 10.1038/s41467-024-44688-3 (PMC10776856; doi:10.1038/s41467-024-44688-3)
Supplement: Supplementary file 1 — Supplementary Information [file 41467_2024_44688_MOESM1_ESM.pdf]

LFA-1 nanoclusters integrate TCR stimulation strength to tune T-cell cytotoxic activity

Lacouture *et al.*

Supplementary material content:

- Supplementary notes

    Estimation of the probability that observed LFA-1 nanoclusters are density fluctuations

    Simulation of STED images

- Supplementary methods

    dSTORM and SIM microscopy

- Supplementary references
- Supplementary Figs. 1 to 9

Supplementary notes

Estimation of the probability that observed LFA-1 nanoclusters are density fluctuations

We model the synapse as a $q\times q$ lattice of elementary membrane domains that may or may not contain a protein cluster. We denote by λ the mean number of proteins in each elementary domain, $\lambda = N/q^2$, where N is the total number of proteins of the species under interest. Without inter-protein interactions, the probability of observing n proteins in a given elementary domain is given by the Poisson distribution

$$P(n) = e^{-\lambda}\frac{\lambda^n}{n!} \qquad (1)$$

The probability that a given elementary domain is identified as a cluster containing *at least* p proteins reads $P_>(p) = \sum_{n=p}^{\infty} P(n)$. Finally, the probability that among the q^2 elementary membrane domains, a number k comprised between $k_{\min}$ and $k_{\max}$ are clusters containing at least p proteins is

$$P(k_{\min}, k_{\max}) = \sum_{k=k_{\min}}^{k_{\max}} \binom{q^2}{k} [P_>(p)]^k [1 - P_>(p)]^{\,q^2-k} \qquad (2)$$

assuming that all domains are independent. This is only an approximation since the total number of available proteins is limited by N.

Let us choose very unfavourable parameter values to estimate a reliable lower bound of this probability in the present experimental context. Since an elementary domain has a typical lateral size of 100 nm and the synapse is roughly 10 µm wide, we choose $q = 100$. Furthermore, we assume that $N = 50000$[*] so that $\lambda = 5$. A cluster contains ≥ 10 proteins ($p = 10$), i.e., only twice as many proteins as the average value λ (which is an extremely unfavorable choice), so that $P_>(10) \simeq 0.03$. Finally, if $k_{\min} = 900$ and $k_{\max} = 1100$, we get $P(k_{\min}, k_{\max}) \sim 10^{-179}$. If, more realistically, $N = 10000$ and so that $\lambda = 1$, then $P(k_{\min}, k_{\max}) \sim 10^{-4988}$.

It is thus extremely unlikely that the observed clusters are just random density fluctuations of an otherwise homogeneous distribution.

[*] We observe $\sim$1000 clusters containing $\sim$10 proteins each, and they account for about 20% of the total fluorescence.

Simulation of STED images

We simulated artificial STED images as follows. An image is composed of $1024{\times}1024$ pixels (pixels of 19 nm side length in real images). Assuming again N proteins in this "membrane", each of them appears as a PSF approximated by a Gaussian of standard deviation σ (the waist is about 2.35σ). The background is constituted of a fraction φ of randomly and homogeneously distributed proteins, whereas the remaining fraction 1-φ is grouped in nanoclusters. Each cluster contains a random number n of proteins randomly distributed inside a region chosen as a rectangle (for simplicity sake) of random sides $l_x{\times}l_y$.

An example of such an image is shown in Supplementary Fig. 9. With about 1000 clusters, it displays 2.5 clusters per µm^2, as observed in a part of the real cells in this work. The total integrin density is about 250 protein/µm^2, also a realistic value [1]. We tested two PSF waist values: 50 nm ($\sigma = 20$ nm), the typical resolution of our STED setup, as if the proteins where punctual, and twice this value, in order to take into account that each integrin has a finite size and can be flanked by several fluorescent antibodies of about 15 nm. These values of σ correspond to the extreme cases that one can envisage in the experimental context.

Then the Trainable Weka Segmentation algorithm, previously trained on STED images, was used on the artificial images. It accurately detected the nanoclusters, as follows. First of all, we adjusted the intensity of the artificial image so that the brightest objects (the clusters) had approximately the same intensity in the STED images and the artificial ones. After detecting the clusters with the help of the segmentation algorithm, and skipping too small objects (≤ 3 pixels) we used the rectangles utilized above to generate the clusters as ROI, and we checked whether each of them contained a part of a detected cluster or not (Supplementary Fig. 9).

In fact, since the clusters are distributed randomly, some of them necessarily overlap and thus we count them as a single artificial cluster. The total number of initial clusters is 960 and 948 if we take this overlap into account. Among them, 916 were actually detected for $\sigma = 20$ nm and 857 for $\sigma = 40$ nm, that is to say more than 90 % were correctly detected. Note that since both the number of integrins in clusters and the cluster size are random variables, their density is also random, between 370 and 2000 protein/µm^2. The chosen lower bound is not far from the background density of 200 protein/µm^2. It follows that the remaining detected clusters are false positives (59 for $\sigma = 20$ nm and 174 for $\sigma = 40$ nm, i.e., 6 % and

18 % respectively). They result from the background density fluctuations. Accordingly, these false positives generally have a surface about 5 times smaller than the true positives. Naturally, the algorithm performance is poorer when for $\sigma = 40$ nm, because the background is denser, however this value has voluntarily been chosen as a likely unfavourable case.

Supplementary methods

dSTORM and SIM microscopy

In parallel to STED imaging, dSTORM imaging was used to detect, separately, the nanometric scale organization of the closed and open conformations of LFA-1 with AF647-labeled Hi-111, and m24 Abs, using an experimental setup described previously [2]. Nanoclusters were detected by density-based spatial clustering of applications with noise (DBSCAN) analysis [2]. SIM imaging was used to detect the closed and open conformation of LFA-1 in the same cells, after costaining with AF647-labeled Hi-111 and AF568-labeled m24 Abs. SIM acquisition and image reconstruction approaches were done as described previously [2].

Supplementary references

[1] S.W. Moore, P. Roca-Cusachs, and M.P. Sheetz, Stretchy proteins on stretchy substrates: the important elements of integrin-mediated rigidity sensing. Developmental cell 19 (2010) 194-206.

[2] R. Houmadi, D. Guipouy, J. Rey-Barroso, Z. Vasconcelos, J. Cornet, M. Manghi, N. Destainville, S. Valitutti, S. Allart, and L. Dupre, The Wiskott-Aldrich Syndrome Protein Contributes to the Assembly of the LFA-1 Nanocluster Belt at the Lytic Synapse. Cell reports 22 (2018) 979-991.

Lacouture *et al.* – **Supplementary Fig. 1**

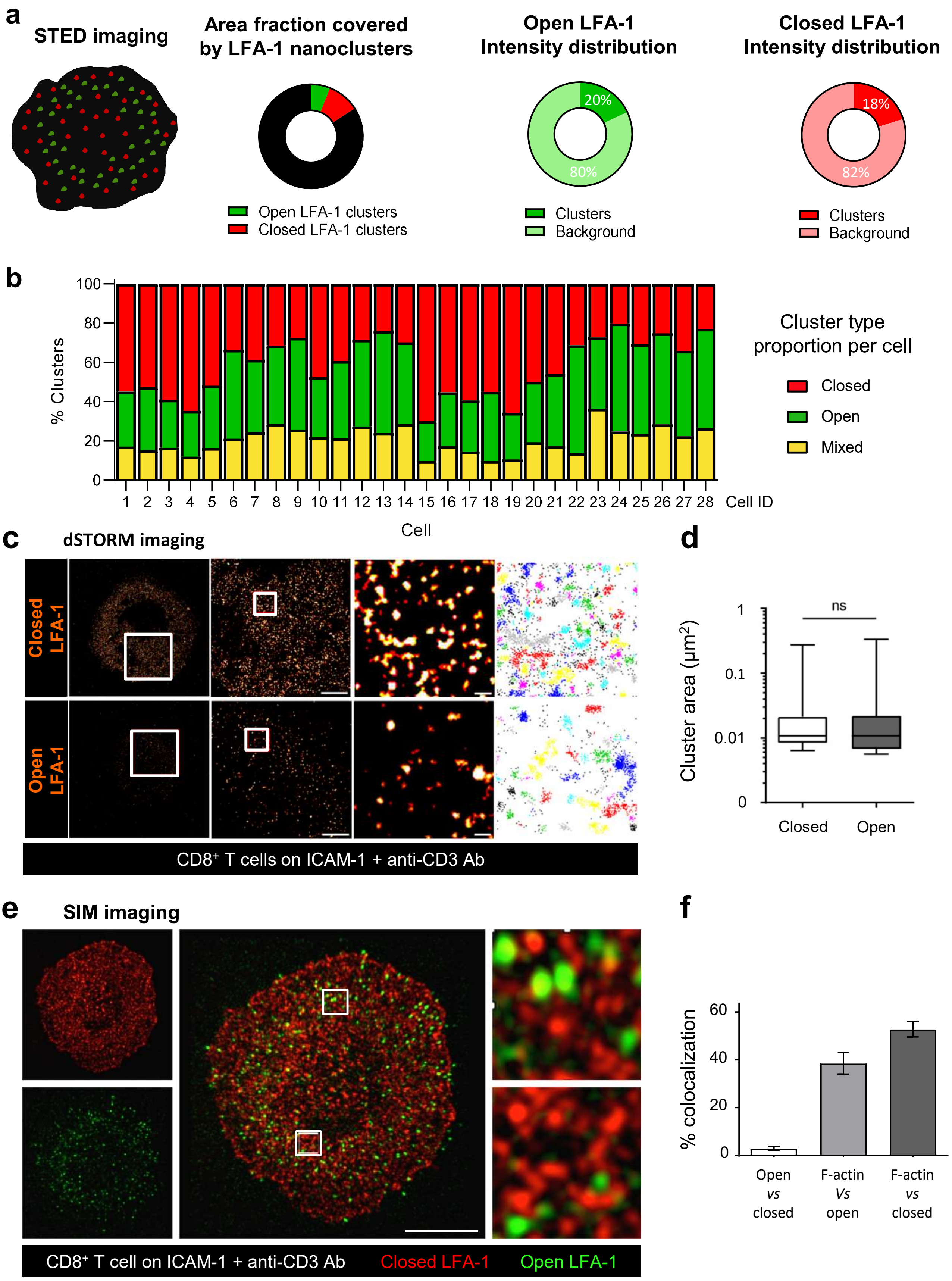

Supplementary Fig. 1 Distribution of LFA-1 conformations at the surface of CD8$^+$ T cells as assessed by STED, dSTORM and SIM imaging modalities. a STED imaging: proportion of the T cell surface (contact plane with ICAM-1 and anti-CD3 Ab coated surface) occupied by the detected open and closed LFA-1 conformation nanoclusters ; proportion of the overall open and closed LFA-1 conformation staining intensities represented by the detected nanoclusters. **b** Distribution of the clusters types as initially detected by applying the Trainable Weka Segmentation tool on STED images from 28 cells from 2 independent experiments ; average proportions : 42% closed conformation clusters, 38% open conformation clusters and 20% mixed conformation clusters. **c** dSTORM imaging of the adhesion plane of 2 representative CD8$^+$ T cells spreading on ICAM-1 and anti-CD3 Ab. Closed (top raw) and open (bottom raw) conformations of LFA-1 were revealed with Hi-111 and m24 Ab, respectively. From left to right, TIRF image, dSTORM reconstruction, first level of zoom, second level of zoom and corresponding DBSCAN detection of clusters. Scale bars: 5 µm (whole cell), 2 µm (zoom 1) and 0.2 µm (zoom 2). **d** Distribution of closed and open conformation nanocluster areas, as detected by dSTORM imaging and DBSCAN processing. Mean and SEM of cluster sizes extracted from 6 cells per staining ; ns: non significative (two-tailed Mann-Whitney non-parametric t test). Box plots indicate median (middle line), 25th, 75th percentile (box) and 5th and 95th percentile (whiskers) **e** SIM imaging of the adhesion plane of one representative CD8$^+$ T cells spreading on ICAM-1 and anti-CD3 Ab. Closed (red) and open (green) conformations of LFA-1 were revealed by costaining with Hi-111 and m24 Abs. Scale bar: 5 µm. **f** Colocalization of the 2 LFA-1 conformations was assessed by calculating the thresholded Mander's coefficient B. Colocalization of each of the LFA-1 conformations with F-actin (cells were also stained with fluorescent phalloidin) was assessed in parallel. Mean and SEM of Mander's coefficient extracted from 8 cells.

Lacouture *et al.* **Supplementary Fig. 2**

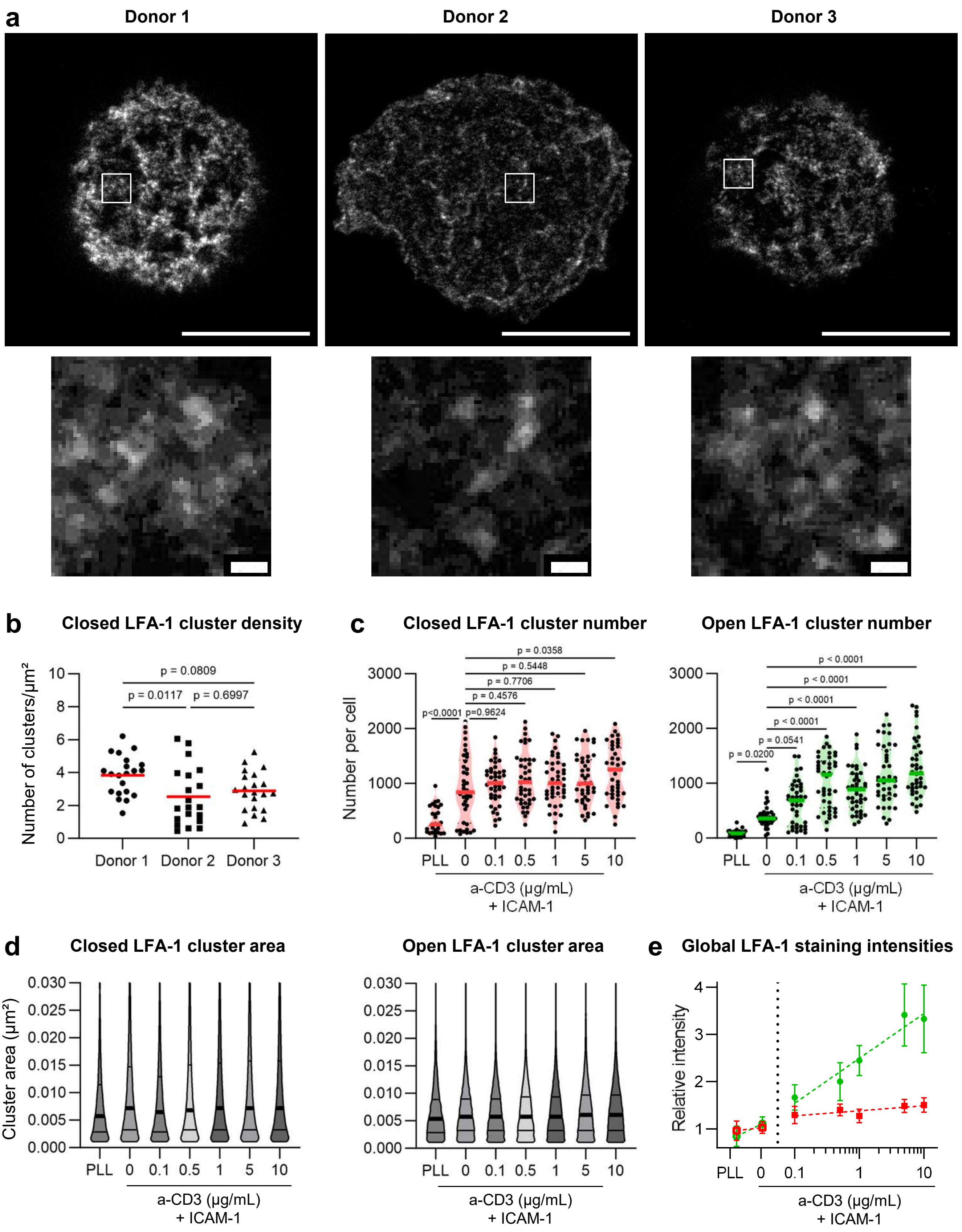

Supplementary Fig. 2 LFA-1 nanocluster numbers as detected by STED imaging. a STED images of representative $CD8^+$ T cells fixed and stained with Hi-111 Ab (closed conformation LFA-1) before being deposited on a glass slide. Scale bars: 5 µm (whole cell) and 0.2 µm (ROI). **b** Density of closed LFA-1 nanoclusters detected by machine-learning algorithm at the surface of unstimulated $CD8^+$ T cells. Significance tests are parametric one-way ANOVA. The number of cells studied per donor are n = 21, 20, 21. The graph shows the results of 2 independent experiments. **c**-**e** The graphs show the results of 3 independent experiments, including T cells from 2 donors. The number of cells studied per condition are n = 27, 38, 40, 42, 41, 42, 39. **c** Number of nanoclusters composed by closed (left) and open (right) LFA-1 molecules, detected at the adhesion plane in individual cells stimulated with the indicated conditions. Solid coloured lines represent the median. The significance tests are parametric one-way ANOVA. **d** Closed (left) and open (right) LFA-1 cluster area. Solid coloured lines represent the median. **e** Relative intensity (compared to ICAM-1 condition) of closed LFA-1 (Hi- 111 Ab staining, red) and open LFA-1 (m24 Ab staining, green) at the adhesion plane of $CD8^+$ T cells spreading on indicated conditions. Mean and SEM are shown.

Lacouture *et al.* **Supplementary Fig. 3**

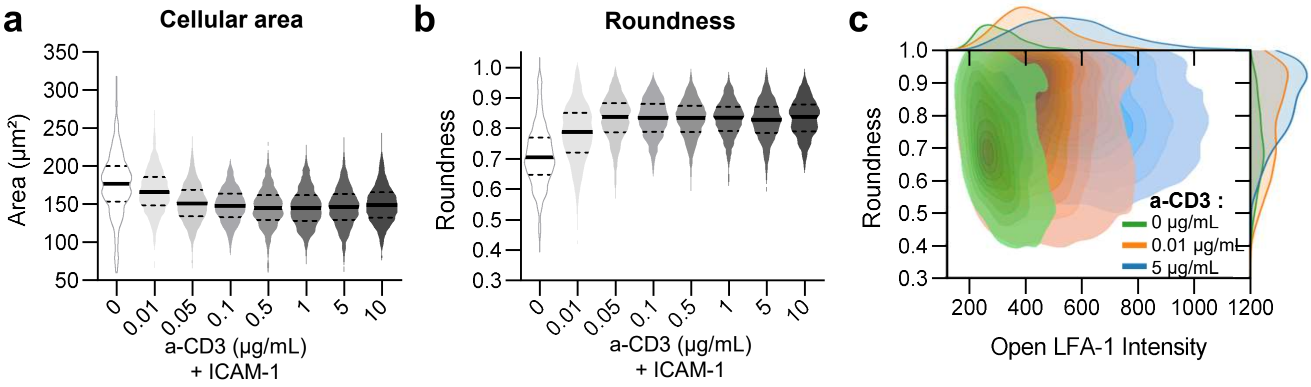

Supplementary Fig. 3 Morphodynamic features of 2D synapses. a Area of cells adhering to ICAM-1 (2 µg/mL) and the indicated concentrations of anti-CD3 Ab. The bars represent the median (middle line), 25th and 75th percentile. The violin plots aggregate the data of 4 independent experiments, each including 2 replicates of the same donor. **b** Roundness of cells adhering to ICAM-1 (2 µg/mL) and the indicated concentrations of anti-CD3 Ab. The bars represent the median (middle line), 25th and 75th percentile. The violin plots aggregate the data of 4 independent experiments, each including 2 replicates of the same donor. **c** Contour plot representing the distribution of the roundness (Y axis) and open LFA-1 staining intensity (X axis) of individual cells. One representative experiment with duplicates among four independent experiments is shown.

Lacouture *et al.* **Supplementary Fig. 4**

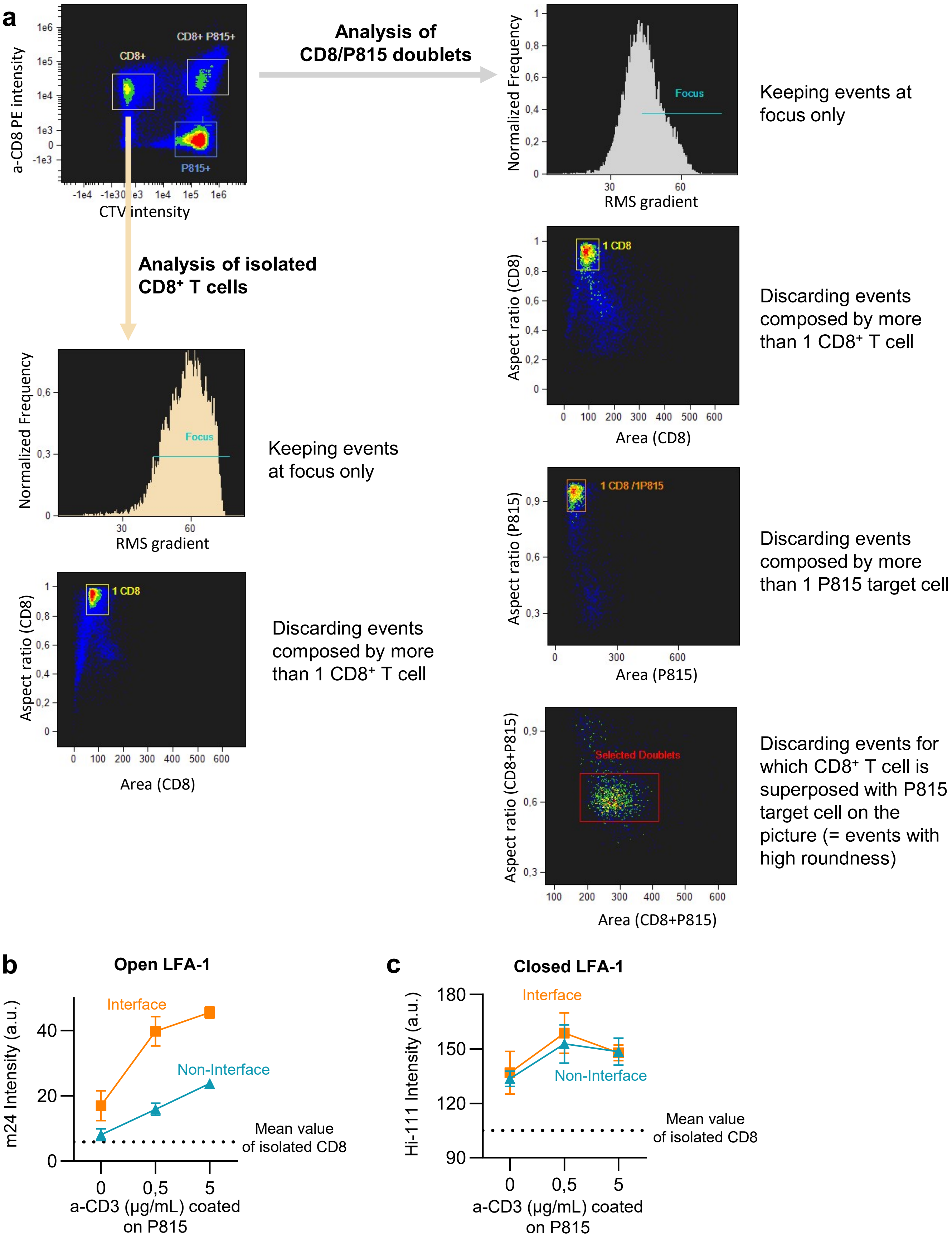

Supplementary Fig. 4 Synaptic enrichment of LFA-1 in conjugates of CD8$^+$ T cells with target cells. a Flow imaging analytical pipeline based on an experiment with P815 target cells coated with 5 µg/mL anti-CD3 Ab. **b, c** Intensities of m24 Ab (**b**) and Hi-111 Ab (**c**) at the interface (orange) and at the non-interface (blue) of CD8$^+$ T cells in contact with P815 target cells coated with the indicated concentrations of anti-CD3 Ab. The dotted line represents the mean intensity of isolated CD8$^+$ T cells. The symbols are the mean of 2 independent experiments and the error bar represents the SEM.

Lacouture *et al.* **Supplementary Fig. 5**

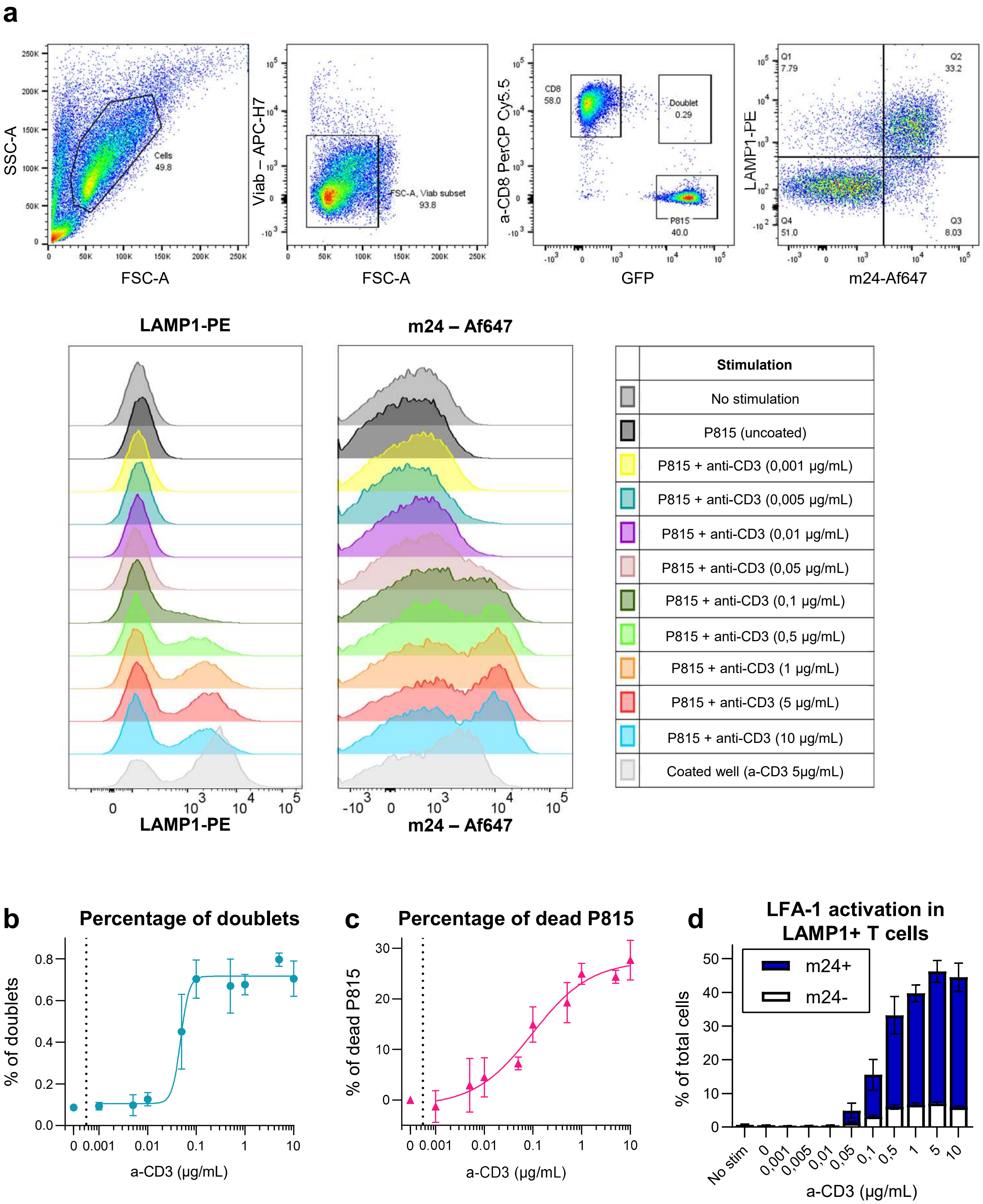

Supplementary Fig. 5 LFA-1 activation and degranulation. **a** Top : example of gating strategy. Bottom : surface expression of LAMP1 (left) and open conformation LFA-1 (m24 Ab, right) in CD8 $^{+}$ T cells stimulated by P815 cells coated with the indicated concentrations of anti-CD3 Ab, as assessed by flow cytometry. One representative experiment among three independent experiments is shown. **b** Percentage of doublets, defined as events detected by flow cytometry as GFP^{+}/CD8^{+}. Mean and SEM of 3 independent experiments. **c** Percentage of dead P815 target cells compared to the condition without anti-CD3 Ab coating. Mean and SEM of 3 independent experiments. **d** Among the total number of CD8^{+} T cells, proportion of cells that are LAMP1+/ m24+ or LAMP1+/m24-. Mean and SEM of 3 independent experiments.

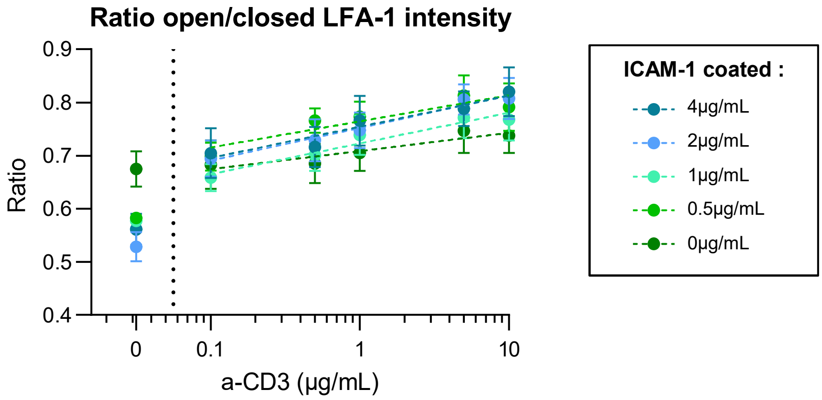

Supplementary Fig. 6 Supplementary Fig.5: Ratio open/closed LFA-1. Ratio open/closed LFA-1 intensity (Hi-111 staining) of cells adhering to a coating made with various ICAM-1 and anti-CD3 Ab concentrations, at the adhesion plane. The symbols represents the mean of 3 independent experiments, each including 2 duplicates, the errors bars are the SEM. Dotted lines represents linear regression on the mean values type a*log[a-CD3 concentration] + b.

Lacouture *et al.* **Supplementary Fig. 7**

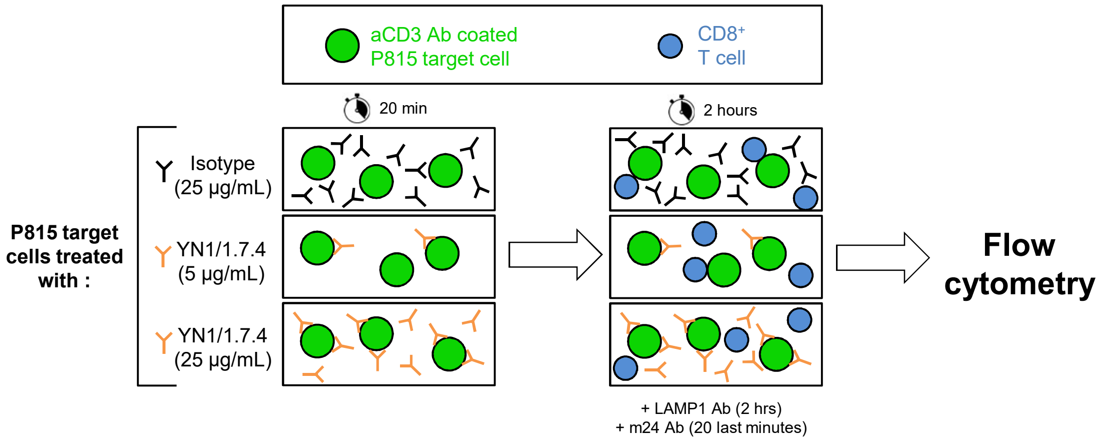

Supplementary Fig. 7 Influence of ICAM-1 availability on killing and contact frequency with target cells. Graphical representation of the flow cytometry based assay to assess concomitantly LFA-1 conformational activation and LAMP-1 surface exposure. Where indicated targets cells were pretreated with an ICAM-1 blocking Ab (YN1.7.4) or isotype control.

Lacouture *et al.* **Supplementary Fig. 8**

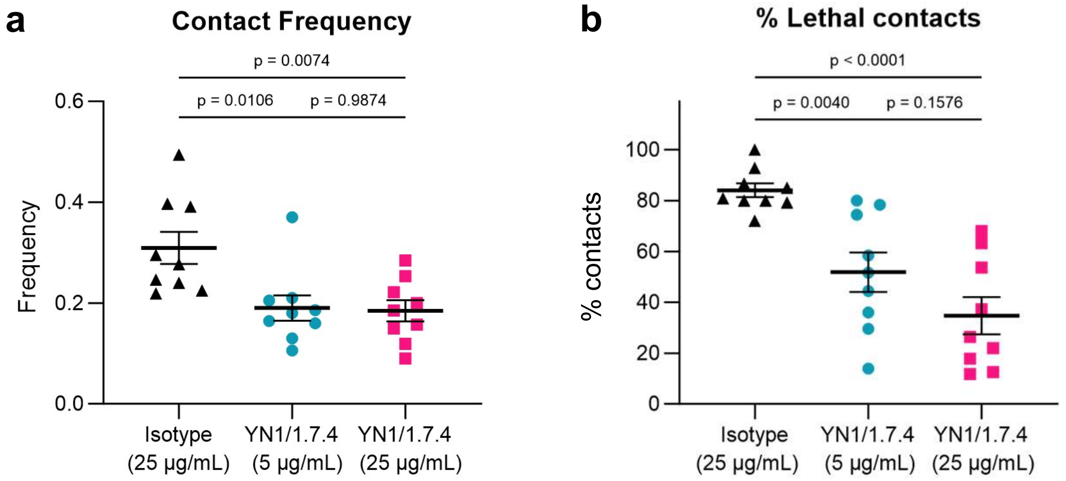

Supplementary Fig. 8 Influence of ICAM-1 availability on killing and contact frequency with target cells. a Live microscopy determination of contact frequency between anti-CD3 Ab (1 µg/mL) coated P815 cells and CD8+ T cells. The plot presents the mean and SEM of three independent experiments, each including 3 fields of view. The statistical tests are one-way ANOVA (Tukey's multiple comparisons tests). The bars represent the median (middle line), 25th and 75th percentile. **b** Live microscopy determination of killing of anti-CD3 Ab (1 µg/mL) coated P815 cells by CD8+ T cells. The plot presents the mean and SEM of three independent experiments, each including 3 fields of view. The statistical tests are one-way ANOVA (Tukey's multiple comparisons tests). The bars represent the median (middle line), 25th and 75th percentile.

Lacouture *et al.* **Supplementary Fig. 9**

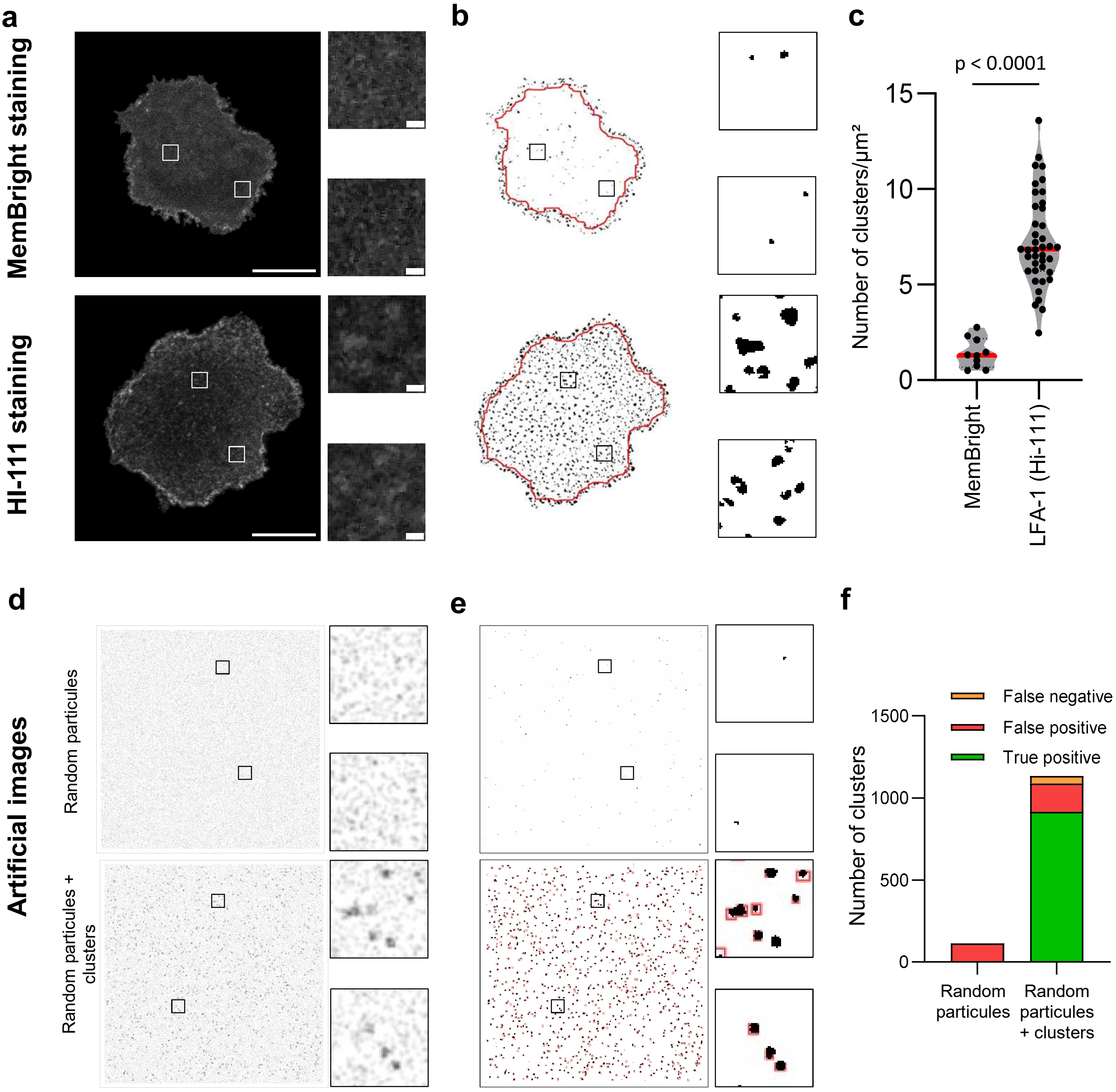

Supplementary Fig. 9 Validation of the nanocluster detection algorithm. a STED images of the adhesion plane of representative CD8$^+$ T cells spreading on ICAM-1/anti-CD3 Ab and staining with MemBright (up) or LFA-1 Hi-111 Ab (down). Scale bar: 5 µm on whole cell, 0,2 µm on zooms. **b** LFA-1 clusters as detected by the trainable Weka segmentation algorithm applied to the STED image presented in a. Due to MemBright accumulation at cellular edges, the studied area (inside the red line) was reduced in order to mask the edges (20 erosion steps, each representing one pixel layer, from the initially detected cell perimeter). **c** Density of clusters detected for the MemBright (n= 10 cells) and Hi-111 Ab (n= 40 cells) inside the studied zone defined in B. Red line represent the median. An unpaired two-sided t-test significance test was applied, $p<0.0001$. **d** in silico generated image. Examples of 1024 × 1024 pixels virtual STED images + zooms of 60x60 pixels. Parameter values are N = 10000, ϕ =0.8, σ = 20 nm (waist of 50 nm), $n \in [12,21]$ and $lx,ly \in 5,9$ pixels. Top : randomly distributed proteins (grey small dots). Bottom : randomly distributed proteins of which 20% are organized in ~1000 clusters (darkest and bigger dots). **e** Images shown in D (+ zooms) were processed with the cluster detection algorithm. In black ROI the clusters detected by the algorithm and in red ROI, the position of simulated clusters (bottom pictures only). **f** Quantification of the detected clusters. True positive = the algorithm detected a cluster where the simulation actually generated one ; False positive = the algorithm detected a cluster where the simulation did not generated one (could be a "natural cluster" created by the fact that some randomly distributed particles are placed near each others) ; False negative = the algorithm did not detected a cluster where the simulation generated one.
