# Supplementary material for: LFA-1 nanoclusters integrate TCR stimulation strength to tune T-cell cytotoxic activity
Source: Nat Commun. 2024 Jan 9;15:407. doi: 10.1038/s41467-024-44688-3 (PMC10776856; doi:10.1038/s41467-024-44688-3)
Supplement: Supplementary file 3 — Description of Additional Supplementary Files [file 41467_2024_44688_MOESM3_ESM.pdf]

Description of Additional Supplementary Files

Supplementary Movie 1: Dynamics of interactions and cytotoxic activity.

Representative movie displaying the time-lapse recording of CD8+ T cells (green) interacting with P815 target cells (unstained) precoated with 1 µg/mL anti-CD3 Ab. Target cell death is monitored through the influx of propidium iodide (red). Time resolution is 1 image per min. Time stamp is hr:min. Scale bar is 10 µm.
