# Supplementary material for: LFA-1 nanoclusters integrate TCR stimulation strength to tune T-cell cytotoxic activity
Source: Nat Commun. 2024 Jan 9;15:407. doi: 10.1038/s41467-024-44688-3 (PMC10776856; doi:10.1038/s41467-024-44688-3)
Supplement: Supplementary file 5 — Reporting Summary [file 41467_2024_44688_MOESM5_ESM.pdf]

nature portfolio

Corresponding author(s): Loïc Dupré and Nicolas Destainville

Last updated by author(s): Nov 10, 2023

Reporting Summary

Nature Portfolio wishes to improve the reproducibility of the work that we publish. This form provides structure for consistency and transparency in reporting. For further information on Nature Portfolio policies, see our Editorial Policies and the Editorial Policy Checklist.

Statistics

For all statistical analyses, confirm that the following items are present in the figure legend, table legend, main text, or Methods section.

n/a	Confirmed	
☐	☒	The exact sample size (*n*) for each experimental group/condition, given as a discrete number and unit of measurement
☐	☒	A statement on whether measurements were taken from distinct samples or whether the same sample was measured repeatedly
☐	☒	The statistical test(s) used AND whether they are one- or two-sided<br>*Only common tests should be described solely by name; describe more complex techniques in the Methods section.*
☒	☐	A description of all covariates tested
☒	☐	A description of any assumptions or corrections, such as tests of normality and adjustment for multiple comparisons
☐	☒	A full description of the statistical parameters including central tendency (e.g. means) or other basic estimates (e.g. regression coefficient) AND variation (e.g. standard deviation) or associated estimates of uncertainty (e.g. confidence intervals)
☒	☐	For null hypothesis testing, the test statistic (e.g. *F*, *t*, *r*) with confidence intervals, effect sizes, degrees of freedom and *P* value noted<br>*Give P values as exact values whenever suitable.*
☒	☐	For Bayesian analysis, information on the choice of priors and Markov chain Monte Carlo settings
☒	☐	For hierarchical and complex designs, identification of the appropriate level for tests and full reporting of outcomes
☒	☐	Estimates of effect sizes (e.g. Cohen's *d*, Pearson's *r*), indicating how they were calculated

Our web collection on statistics for biologists contains articles on many of the points above.

Software and code

Policy information about availability of computer code

Data collection	Commercial software used to collect data: BD FACSDiva v9.0, Harmony high-content analysis software 5.0
Data analysis	Commercial software used to analyze data: Prism 9.4.0, FlowJo 10.9, Fiji, Harmony high-content analysis software 5.0<br>A custom code was used to generate artificial STED images. It can be accessed with the following link: https://doi.org/10.5281/zenodo.10084714

For manuscripts utilizing custom algorithms or software that are central to the research but not yet described in published literature, software must be made available to editors and reviewers. We strongly encourage code deposition in a community repository (e.g. GitHub). See the Nature Portfolio guidelines for submitting code & software for further information.

Data

Policy information about availability of data

All manuscripts must include a data availability statement. This statement should provide the following information, where applicable:

- Accession codes, unique identifiers, or web links for publicly available datasets
- A description of any restrictions on data availability
- For clinical datasets or third party data, please ensure that the statement adheres to our policy

The raw data collected to elaborate the figures of the paper are shared via FigShare. All other source data (.tiff images and .fcs flow cytometry files) are available from the corresponding authors upon request. Data are located in controlled access data storage at the INSERM Infinity Institute and the Theoretical Physics

Laboratory of the University of Toulouse.

Research involving human participants, their data, or biological material

Policy information about studies with human participants or human data. See also policy information about sex, gender (identity/presentation), and sexual orientation and race, ethnicity and racism.

Reporting on sex and gender	Due to the limited sample size (3 donors), no sex or gender issue was considered in this study.
Reporting on race, ethnicity, or other socially relevant groupings	Not applicable.
Population characteristics	Due to the limited sample size (3 donors), no population issue was considered in this study.
Recruitment	The donors are volunteers donating blood to the Etablissement Francais du Sang - Occitanie (Toulouse, France). There were fully anonymized.
Ethics oversight	Comité de Protection des Personnes (CPP) Sud-Ouest et Outre-Mer I/II.

Note that full information on the approval of the study protocol must also be provided in the manuscript.

Field-specific reporting

Please select the one below that is the best fit for your research. If you are not sure, read the appropriate sections before making your selection.

[x] Life sciences [ ] Behavioural & social sciences [ ] Ecological, evolutionary & environmental sciences

For a reference copy of the document with all sections, see nature.com/documents/nr-reporting-summary-flat.pdf

Life sciences study design

All studies must disclose on these points even when the disclosure is negative.

Sample size	The main biological findings established with T cells from one donor were confirmed with T cells from two other donors. Such sample size is usually sufficient in ensuring that the basic cell biology observations are robust.
Data exclusions	No data were excluded.
Replication	The experiments reported in the study included technical replicates, as stated in the figure legends. They were systematically reproduced at least once through independent experiments.
Randomization	Given the sample size, randomization is not relevant.
Blinding	Not relevant since we did not expect a priori any sort of differences among the donors.

Reporting for specific materials, systems and methods

We require information from authors about some types of materials, experimental systems and methods used in many studies. Here, indicate whether each material, system or method listed is relevant to your study. If you are not sure if a list item applies to your research, read the appropriate section before selecting a response.

Materials & experimental systems

n/a	Involved in the study	
[ ]	[x]	Antibodies
[ ]	[x]	Eukaryotic cell lines
[x]	[ ]	Palaeontology and archaeology
[x]	[ ]	Animals and other organisms
[x]	[ ]	Clinical data
[x]	[ ]	Dual use research of concern
[x]	[ ]	Plants

Methods

n/a	Involved in the study	
[x]	[ ]	ChIP-seq
[ ]	[x]	Flow cytometry
[x]	[ ]	MRI-based neuroimaging

Antibodies

Antibodies used	anti-CD3 (clone OKT3, Invitrogen ref : 16-0037-85), anti-CD11a Af594 (clone Hi-111, Biolegend ref : 301222), biotin anti-CD11a/CD18 (clone m24, Biolegend ref : 363424), anti-CD11a Af488 (clone Hi-111, Biolegend ref : 301216), anti-CD11a/CD18 Af647 (clone m24,

	Biolegend ref : 363412), anti-CD8 (clone RPAT8, BD Biosciences ref : 555367), anti-LAMP1/CD107a PE (clone H4A3, BD Biosciences ref : 560948), anti-CD8 PerCP Cy5.5 (clone MEM-31, Sysmex ref : AE134275), anti-CD11a FITC (clone TS2/4, Biolegend ref : 350604), anti-CD54 (clone YN1/1.7.4, Biolegend ref : 116102).
Validation	Antibody used have all been validated in previous reports.

Eukaryotic cell lines

Policy information about cell lines and Sex and Gender in Research

Cell line source(s)	The GFP-expressing P815 cell line was previously generated in our laboratory (Houmadi et al., Cell Reports 2018).
Authentication	The cell line was regularly checked for ICAM-1 and GFP expression.
Mycoplasma contamination	Mycoplasma tests were routinely performed on the cell lines used in the study and were negative.
Commonly misidentified lines (See ICLAC register)	No commonly misidentified lines were used (according to ICLAC register).

Plants

Seed stocks	*Report on the source of all seed stocks or other plant material used. If applicable, state the seed stock centre and catalogue number. If plant specimens were collected from the field, describe the collection location, date and sampling procedures.*
Novel plant genotypes	*Describe the methods by which all novel plant genotypes were produced. This includes those generated by transgenic approaches, gene editing, chemical/radiation-based mutagenesis and hybridization. For transgenic lines, describe the transformation method, the number of independent lines analyzed and the generation upon which experiments were performed. For gene-edited lines, describe the editor used, the endogenous sequence targeted for editing, the targeting guide RNA sequence (if applicable) and how the editor was applied.*
Authentication	*Describe any authentication procedures for each seed stock used or novel genotype generated. Describe any experiments used to assess the effect of a mutation and, where applicable, how potential secondary effects (e.g. second site T-DNA insertions, mosiacism, off-target gene editing) were examined.*

Flow Cytometry

Plots

Confirm that:

- [x] The axis labels state the marker and fluorochrome used (e.g. CD4-FITC).
- [x] The axis scales are clearly visible. Include numbers along axes only for bottom left plot of group (a 'group' is an analysis of identical markers).
- [x] All plots are contour plots with outliers or pseudocolor plots.
- [x] A numerical value for number of cells or percentage (with statistics) is provided.

Methodology

Sample preparation	the T cells has been isolated from human blood sample with Ficoll Paques, and cultivated as detailed in the Materials and Methods section of the manuscript.
Instrument	BD LSRFortessa™ X-20 Cell Analyzer.
Software	Diva (acquisition) and Flowjo (analysis).
Cell population abundance	At least 10 000 cells per condition.
Gating strategy	Gating strategy is shown in the Supplementary Fig. 4 and 5A.

- [x] Tick this box to confirm that a figure exemplifying the gating strategy is provided in the Supplementary Information.
